# Novel Insights into the Roles of Rho Kinase in Cancer

**DOI:** 10.1007/s00005-015-0382-6

**Published:** 2016-01-02

**Authors:** Lei Wei, Michelle Surma, Stephanie Shi, Nathan Lambert-Cheatham, Jianjian Shi

**Affiliations:** Riley Heart Research Center, Herman B Wells Center for Pediatric Research, Department of Pediatrics, Indiana University, School of Medicine, R4 Building, Room 332, 1044 West Walnut Street, Indianapolis, IN 46202-5225 USA; Department of Cellular and Integrative Physiology, Indiana University, School of Medicine, 1044 West Walnut Street, R4-370, Indianapolis, IN 46202-5225 USA

**Keywords:** Rho kinase, Rho kinase pan-inhibitor, ROCK isoform-specific inhibitor, Cancer therapy

## Abstract

Rho-associated coiled-coil kinase (ROCK) is a major downstream effector of the small GTPase RhoA. The ROCK family, consisting of ROCK1 and ROCK2, plays a central role in the organization of the actin cytoskeleton, and is involved in a wide range of fundamental cellular functions such as contraction, adhesion, migration, proliferation, and apoptosis. Since the discovery of effective inhibitors such as fasudil and Y27632, the biological roles of ROCK have been extensively explored in numerous diseases, including cancer. Accumulating evidence supports the concept that ROCK plays important roles in tumor development and progression through regulating many key cellular functions associated with malignancy, including tumorigenicity, tumor growth, metastasis, angiogenesis, tumor cell apoptosis/survival and chemoresistance as well. This review focuses on the new advances of the most recent 5 years from the studies on the roles of ROCK in cancer development and progression; the discussion is mainly focused on the potential value of ROCK inhibitors in cancer therapy.

## Introduction

Rho-associated coiled-coil kinase (ROCK) is one of the best characterized effectors of the small GTPase RhoA and belongs to the AGC family of serine/threonine protein kinases, which also includes protein kinases A, G, and C (PKA, PKG, PKC) (Ishizaki et al. 1996; Leung et al. 1996; Matsui et al. 1996; Nakagawa et al. 1996). The ROCK family consists of two isoforms, ROCK1 and ROCK2, sharing 65 % overall homology and 92 % homology in the kinase domain. Both kinases contain a catalytic kinase domain at the N terminus followed by a central coiled-coil domain, which includes the Rho-binding domain (RBD), and a C-terminal pleckstrin-homology (PH) domain. The primary roles of the ROCK family in the organization of actin cytoskeleton have been well established, and they are involved in a wide range of fundamental cellular functions such as contraction, adhesion, migration, proliferation, and apoptosis (Amano et al. 2010a; Julian and Olson 2014; Shi and Wei 2007; Street and Bryan 2011). Since the discovery of the ROCK family, the Rho/ROCK signaling pathway has attracted much attention in various research fields, and more than 10,000 articles have been published; in particular, about 2000 articles are focused on Rho/ROCK function in cancer. Accumulating evidence from basic and clinical studies supports the concept that ROCK could be a potential therapeutic target for diverse disorders, including cardiovascular disorders, neurologic disorders, metabolic disorders, and cancers (Huang et al. 2013; Knipe et al. 2015; Morgan-Fisher et al. 2013; Rath and Olson 2012; Sawada and Liao 2014; Shi and Wei 2013; Watzlawick et al. 2014).

The initiation and progression of cancer are multistep events involving cellular transformation, tumor growth, neovascularization, invasion, and metastasis. The roles of ROCK in various cancer processes have been extensively explored with a particular attention focused on tumor cell motility, invasion, and metastasis (Chen et al. 2014; Kale et al. 2015; Mali et al. 2014; Mardilovich et al. 2012; Matsuoka and Yashiro 2014; Morgan-Fisher et al. 2013; Rath and Olson 2012; Schofield and Bernard 2013). In these studies, Y27632 (Uehata et al. 1997) and fasudil (Asano et al. 1987), relatively selective ROCK inhibitors which target the ATP-dependent kinase domain of ROCK1 and ROCK2, have been extensively used in dissecting their roles in cellular signaling and animal disease models. However, these inhibitors inhibit ROCK1 and ROCK2 with similar potency (Breitenlechner et al. 2003; Davies et al. 2000; Ishizaki et al. 2000; Uehata et al. 1997), and cannot be used to distinguish the functional differences between ROCK1 and ROCK2. The specific disruption of each ROCK isoform in mice offers a unique opportunity to analyze in vivo physiological and pathological functions of ROCK1 and ROCK2. This review focuses on the new advances in exploring the roles of ROCK signaling in cancer biology from the past 5 years and the discussion mainly focuses on the potential value of ROCK inhibitors as a novel anti-cancer approach in clinical therapy. Recent findings derived from targeting ROCK1 and ROCK2 by genetic approaches, short interfering RNA (siRNA) or short hairpin RNA (shRNA)-based gene silencing techniques, are also covered in the review.

## Overview of ROCK Signaling Pathway

### Substrates of ROCK

ROCK1 and ROCK2 share more than 30 immediate downstream substrates due to the high degree of homology in their kinase domains, and many of them are related to the regulation of actin cytoskeleton and cell morphology (Amano et al. 2010a; Morgan-Fisher et al. 2013; Schofield and Bernard 2013; Shi and Wei 2007). The canonical substrates of ROCK include myosin light chain (MLC) (Amano et al. 1996; Kureishi et al. 1997), MLC phosphatase (Kawano et al. 1999; Kimura et al. 1996), LIM kinase (Amano et al. 2001; Lin et al. 2003; Maekawa et al. 1999; Ohashi et al. 2000; Sumi et al. 2001), ezrin/radixin/moesin (Matsui et al. 1998), and adducin (Fukata et al. 1999). The consensus amino acid sequences which are phosphorylated on these substrates are R/KXS/T or R/KXXS/T (Kawano et al. 1999; Sumi et al. 2001). However, these substrates can also be phosphorylated by other serine-threonine kinases such as MLC kinase and several other members of the AGC kinase family (Pearce et al. 2010; Prudnikova et al. 2015). The ROCK/MLC phosphatase/MLC and ROCK/LIM kinase/cofilin pathways are profoundly involved in the regulation of actin filament dynamics that are important for the regulation of cell contractility, motility, and morphology. ROCK promotes actomyosin contractility through increasing MLC phosphorylation, and stabilizes actin filaments through LIM kinase activation, resulting in cofilin phosphorylation and thereby inhibiting its actin-depolymerization activity.

Novel ROCK substrates are discovered constantly and added to the list of the large cohort of substrates. A novel substrate is elongation initiation factor-1-α-1, which was found using a mutant ROCK2 containing a modified ATP pocket to allow the use of selective ATP analogs, which are not efficiently utilized by other protein kinases (Couzens et al. 2014). Likewise, synthetic peptide substrates have been developed for ROCK2, which can be used in mechanistic studies and drug development (Kang et al. 2011). In the context of tumor cell migration, FilGAP, a GTPase activating protein, is phosphorylated by ROCK in the ameboid migration of carcinoma cells (Saito et al. 2012). Moreover, a recent proteomic approach has identified 121 proteins as candidate substrates (Amano et al. 2010b; Nishioka et al. 2012, 2015). Given the abundance of ROCK substrates which are functionally diverse proteins, ROCK proteins are involved in a wide range of fundamental cellular functions such as contraction, adhesion, migration, proliferation, and apoptosis as discussed below.

### Regulation of ROCK Activity

ROCK exhibits auto-inhibitory activity (Amano et al. 1999); in its inactive form, the C-terminal PH domain and RBD of ROCK interact with the kinase domain, which forms an auto-inhibitory loop. ROCK activation occurs in several ways: through interaction with common activators, via alteration of subcellular localization, and by interaction with isoform-specific regulatory molecules. The small Rho GTPases, including RhoA, RhoB, and RhoC, are the most deliberate ROCK regulators. Activated Rho directly interacts with the RBD of ROCK and induces a conformational change initiating interactions of serine/threonine kinases with selective substrates (Blumenstein and Ahmadian 2004; Fujisawa et al. 1996; Ishizaki et al. 1996; Leung et al. 1996; Matsui et al. 1996; Nakagawa et al. 1996). ROCK activity can also be modulated through interaction of C-terminal PH domain with lipid mediators and with the plasma membrane (Feng et al. 1999; Fu et al. 1998; Shirao et al. 2002; Wen et al. 2008), auto-phosphorylation through dimerization (Chuang et al. 2012; Couzens et al. 2009; Doran et al. 2004; Dvorsky et al. 2004; Garg et al. 2008; Jacobs et al. 2006; Yamaguchi et al. 2006), and proteolytic cleavage of the inhibitory C-terminal domain (Coleman et al. 2001; Sebbagh et al. 2001, 2005).

Other than common regulators such as RhoA/RhoB/RhoC, ROCK1, and ROCK2 can also be individually activated or inhibited by a number of positive or negative regulators. The small GTP-binding protein RhoE interacts with the N-terminal region of ROCK1 (amino acids 1–420) and prevents Rho binding to RBD (Komander et al. 2008; Ongusaha et al. 2006; Riento et al. 2003). Phosphoinositide-dependent protein kinase 1 selectively promotes ROCK1 membrane translocation and blocks its association with RhoE (Pinner and Sahai 2008). ROCK1 is cleaved by caspase-3 at the cleavage site DETD1113 during apoptosis, but this site is not present in ROCK2 (Coleman et al. 2001; Sebbagh et al. 2001). On the other hand, during cytotoxic lymphocyte granule-induced cell death, human ROCK2 can be cleaved by the proapoptotic protease granzyme B at IGLD1131 site; however, the site is not present in ROCK1 (Sebbagh et al. 2005). Human ROCK2, but not ROCK1, can be activated by caspase-2-dependent cleavage in endothelial cells in response to thrombin, though the cleavage site remains to be identified (Sapet et al. 2006). ROCK2 activity can be negatively regulated through interacting with coronin1A/B via its PH domain (Rana and Worthylake 2012) and with collapsin response-mediator protein 2 (Yoneda et al. 2012), or positively regulated through interacting with nucleophosmin (Ferretti et al. 2010). Other studies have revealed that ROCK1 and ROCK2 are phosphorylated by different kinases at multiple sites which could apply various influences on their activities (Du and Hannon 2004; Lee et al. 2010; Lowery et al. 2007).

Many studies are focused on how the Rho/ROCK pathway is associated with cancer progression. Increased Rho/ROCK activity and/or gene expression have been demonstrated in various types of cancers. In addition, increased expression of ROCK protein or mRNA negatively correlates with patient survival and positively with the more advanced tumor stages and worse prognostics, further supporting a contributory role of ROCK in cancer progression (Abe et al. 2008; Gilkes et al. 2014; Lane et al. 2008; Li et al. 2015b; Wong et al. 2009; Zhang et al. 2015). The factors attributable to the increased Rho/ROCK activity and/or gene expression are context dependent. For example, in responding to the hypoxic microenvironment, hypoxia-inducible factors increase transcription of RhoA and ROCK1 in breast cancer (Gilkes et al. 2014). In some cases, Rho/ROCK activation is part of a downstream signaling cascade: from SMAD4/TGF-β/BMP in colorectal cancer (Voorneveld et al. 2014), vascular endothelial growth factor in cervical cancer (He et al. 2010), androgen in prostate cancer (Schmidt et al. 2012), chemokine receptor 7 activation in metastatic squamous cell carcinoma of the head and neck (Xu et al. 2015), epidermal growth factor (EGF)/EGF receptor in pancreatic cancer (Nakashima et al. 2011), and increased expression of the receptor for activated C-kinase-1, a scaffolding protein, interacting with RhoA to activate RhoA/ROCK pathway in breast cancer (Cao et al. 2011). In some cases, Rho/ROCK activation is due to the loss of an antagonist or inhibitor. In hepatocellular carcinoma, the loss of the antagonist RhoE leads to increased ROCK activity (Ma et al. 2013); in invasive lobular carcinoma, p120-cadherin inhibits the antagonist myosin phosphatase Rho-interacting protein leading to increased Rho/ROCK activity (Schackmann et al. 2011); in pancreatic and liver cancer metastasis, the overexpression of tropomyosin-related kinase B leads to binding and sequestering Rho GDP dissociation inhibitor, subsequently activating Rho and its downstream pathways (Li et al. 2009). Different external molecules also induce Rho/ROCK activation. In nasopharyngeal carcinoma, *N*,*N*′-dinitrosopiperazine, a carcinogen, increases phosphorylation of ezrin via Rho/ROCK and PKC pathways, leading to increased motility and invasion (Tang et al. 2011).

Rho/ROCK activity can also be spatially regulated, in particular, during cancer cell migration. In pancreatic ductal adenocarcinoma, RhoA is spatially regulated to the rear and leading edges of cells, leading to cancer cell invasion (Timpson et al. 2011); in brain and breast tumor cells, a RhoA-specific guanine nucleotide exchange factor, Syx, moves to the membrane, where it activates RhoA and downstream effector Dia1 and suppresses ROCK activity, resulting in polarized cancer cell migration (Dachsel et al. 2013); and NG2, a membrane proteoglycan, promotes amoeboid invasiveness of carcinoma cells through the activation of Rho (Pankova et al. 2012).

### Association Between ROCK Polymorphisms and Cancer Development

To investigate the role of ROCKs in cancer, identifying mutated genes and evaluating their potential risks are important steps. More than 600 somatic coding mutations in both ROCK genes have been identified in human cancer genomes originating from human cancer cell lines and primary tumors (http://cancer.sanger.ac.uk/cosmic/search?q=ROCK1) (http://cancer.sanger.ac.uk/cosmic/search?q=ROCK2). In addition, several thousands of single nucleotide polymorphisms (SNPs) of ROCK1 and ROCK2 genes have been identified (www.ncbi.nlm.nih.gov/snp/?term=ROCK1) (www.ncbi.nlm.nih.gov/snp/?term=ROCK2). The role and impact of these mutations and SNPs in cancer progression remain largely unclear. Recent studies have validated some of the mutations and gene variants, with an emphasis on examining those affecting the coding sequence of ROCK1 and ROCK2, and determining their impact on cancer pathogenesis (Table 1). Three of the somatic ROCK1 mutations, which were identified in human cancers, have been further analyzed to determine their functional impact by molecular and cellular approaches (Lochhead et al. 2010). Among the three mutations, two lead to premature termination of translation at Tyr405 and Ser1126 in primary breast cancers, and one leads to a substitution of proline 1193 with serine in lung carcinoma. All three mutations increase kinase activity attributable to the removal of auto-inhibition, promote contraction, increase motility, and decrease adhesion (Lochhead et al. 2010). Other mutations, located in the coiled-coil domain, may affect dimerization, RhoA binding, and kinase activation, such as Thr431Asn (Kalender et al. 2010), Asp601Val, and Lys1083Met in ROCK2 (Sari et al. 2013).

**Table 1:** ROCK mutations associated with cancers

Isoform	Coding mutation	Domain	Function	Association with cancer	References
ROCK1	Tyr405*	Kinase	Activation	Breast cancer	Lochhead et al. (2010)
	Ser1126*	PH	Activation	Breast cancer	Lochhead et al. (2010)
	Pro1193S	PH	Activation	Lung cancer	Lochhead et al. (2010)
	Val1309*	PH	May increase kinase activity	Colorectal cancer with microsatellite instability	Alhopuro et al. (2012)
ROCK2	Thr431Asn	Coiled-coil	May affect dimerization, Rho binding, and kinase activation	Breast cancer	Kalender et al. (2010)
	Asp601Val	Coiled-coil	May affect dimerization, Rho binding, and kinase activation	Colorectal cancer	Sari et al. (2013)
	Lys1083Met	Coiled-coil	May affect dimerization, Rho binding, and kinase activation	Colorectal cancer	Sari et al. (2013)
	Increased gene copy number (2.8×)		Increased expression/activation	Malignant peripheral nerve sheath tumor	Upadhyaya et al. (2012)

Other studies support a potential contributory role of the somatic mutations to human cancers by analyzing the mutation frequency. Two ROCK1 and five ROCK2 polymorphisms were found significantly associating with colorectal cancer development (Sari et al. 2013). ROCK1 is a candidate gene involved in microsatellite instability (genetic instability due to problems with DNA mismatch repair) also correlated with colorectal cancer development (Alhopuro et al. 2012). Thr431Asn polymorphism of the ROCK2 gene could be a risk factor for the metastases of breast cancer (Kalender et al. 2010). ROCK2 was found to be commonly mutated in non-small cell lung cancer by whole-exome sequencing (Liu et al. 2012a), and its copy number and gene expression were increased in malignant peripheral nerve sheath tumors (Upadhyaya et al. 2012).

### ROCK and MicroRNAs

MicroRNAs (miRNA) are small pieces of RNA found between protein coding genes or embedded within their introns. These pre-miRNA transcripts are processed into one or more mature miRNAs of ~21 to 22 nucleotides which then regulate cell processes by binding to the 3′ untranslated regions (UTR) of targeted messenger RNA, leading to mRNA degradation and decreased translational efficiency. Numerous miRNAs involved in regulating ROCK1 and ROCK2 expression and activity have been identified in cancer tissues. The down-regulation of specific miRNAs is correlated with increased ROCK1 or ROCK2 expression (Table 2). Since the 3′ UTRs of ROCK1 and ROCK2 comprise different sets of miRNA-binding sites, their expressions can be differently regulated by miRNAs. ROCK1 was found to be a target of miR-145 (Wan et al. 2014) and miR-124 (An et al. 2013) in glioma, miR-145 (Li et al. 2014a) and miR-340 (Cai et al. 2014; Zhou et al. 2013) in osteosarcoma, miR-148a (Zheng et al. 2011), miR-135a (Shin et al. 2014) and miR-124 (Hu et al. 2014) in gastric cancer, miR-186 (Cui et al. 2014) and miR-148a (Li et al. 2013b) in non-small cell lung cancer cells, miR146a (Lin et al. 2008) and miR-135a (Kroiss et al. 2015) in prostate cancer, miR-584 (Ueno et al. 2011) in renal cancer, and miR-1280 (Majid et al. 2012) in bladder cancer. Decreased expression of the miRNAs in cancer tissues leads to increased ROCK expression/activity and increased migration, invasion, or proliferation, which can be rescued by either overexpression of the miRNAs or inhibition of ROCK1 by either a ROCK inhibitor (Y27632) or ROCK1 siRNA molecules (An et al. 2013; Hu et al. 2014; Li et al. 2013b, 2014a; Ueno et al. 2011; Zheng et al. 2011; Zhou et al. 2013). It is worth noting that miRNAs also target other transcripts in addition to ROCK1, such as miR-148b and miR-335 targeting multiple genes in breast cancer (Cimino et al. 2013) and in neuroblastoma cells (Lynch et al. 2012), respectively. Another example is miR135a which targets both ROCK1 and ROCK2 as well as numerous genes involved in cellular movement, cellular assembly/organization, and cell morphology in prostate cancer cells (Kroiss et al. 2015).

**Table 2:** Regulation of ROCK1 and ROCK2 by miRNAs

Isoform	MiRNA	Cancer	References
ROCK1	miR-124	Brain	An et al. (2013)
		Gastric	Hu et al. (2014)
	miR-135a	Gastric	Shin et al. (2014)
	miR-145	Bone	Li et al. (2014a)
		Brain	Wan et al. (2014)
	miR-146a	Prostrate	Lin et al. (2008)
	miR-148a	Gastric	Zheng et al. (2011)
		Lung	Li et al. (2013b)
	miR-148b	Breast	Cimino et al. (2013)
	miR-186	Lung	Cui et al. (2014)
	miR-340	Bone	Zhou et al. (2013)
	miR-335	Brain	Lynch et al. (2012)
	miR-584	Renal	Ueno et al. (2011)
	miR-1280	Bladder	Majid et al. (2012)
ROCK2	miR-101	Liver	Zheng et al. (2015)
	miR-124	Liver	Zheng et al. (2012)
	miR-138	Tongue	Jiang et al. (2010)
	miR-139	Liver	Wong et al. (2011)
	miR-200 b/c	Liver	Peng et al. (2013)
ROCK1 and 2	miR-135a	Prostate	Kroiss et al. (2015)

In addition to those miRNAs interacting with ROCK1, there are also miRNAs interacting with ROCK2 in cancers, for instance, miR-139 (Wong et al. 2011), miR-124 (Zheng et al. 2012) and miR-101 (Zheng et al. 2015) in hepatocellular carcinoma, miR-200b/c in cholangiocarcinoma (Peng et al. 2013), and miR-138 in oral cancer (Jiang et al. 2010). The down-regulation of these miRNAs in cancer tissues leads to increased ROCK2 levels and increased invasion, migration, and proliferation. These miRNAs also target other transcripts in addition to ROCK2, such as EZH2 with miR-124 (Zheng et al. 2012) and SUZ12 with miR-200b/c (Peng et al. 2013). Furthermore, there are miRNAs targeting upstream activators of ROCK, for instance, miR-126 targets the upstream RhoA, and its down-regulation in colorectal cancers with metastasis results in the activation of RhoA/ROCK activity (Li et al. 2013c); miR-26a, which is often amplified in glioblastoma, targets the cyclin-dependent kinase-associated phosphatase (KAP, a member of the dual-specificity protein phosphatase family and is able to bind multiple cyclin-dependent kinases), a novel binding partner and activator of ROCK2; down-regulation of KAP leads to decreased ROCK2 activity and this, in turn, increases Rac GTPase-mediated invasion (Li et al. 2015a).

While ROCK mRNAs have been shown to be targeted by miRNA-mediated degradation as mentioned above, recent reports also revealed the effects of ROCK signaling on miRNA expression and function. ROCK1 directly interacts with and stabilizes the oncogene c-Myc protein leading to the increased oncomir miR-17–92 cluster expression in breast cancer and prostate cancer (Liu et al. 2009; Zhang et al. 2014). Inhibition of ROCK activity in vascular tumor-forming endothelial cells was reported to alter the global miRNA expression (Stiles et al. 2013). Moreover, ROCK inhibitors enhance miRNA function by promoting miRNA-mediated degradation of mRNA targets; ROCK inhibitors induce a conformational change of ROCK1 thereby enhancing its binding to the transcription factor, hepatocyte nuclear factor 4 alpha, leading to increased transcription of poly(A)-binding protein-interacting protein 2, which enhances poly(A)-shortening of miRNA-targeted mRNAs and leads to global up-regulation of miRNA function (Yoshikawa et al. 2015). The general reduction in miRNA expression and impaired miRNA processing are commonly observed in human and experimental cancers, suggesting that they may be related to tumorigenesis (Calin and Croce 2006), hence the effects of the ROCK inhibitor-induced increase in global miRNA activity may be valuable for developing novel cancer therapeutics.

## ROCK is a Key Player in Cancer Progression

ROCK and its downstream targets are involved in regulating actin cytoskeleton dynamics, and therefore are responsible for cell migration and motility. In addition, they are implicated in diverse biological processes such as cell junction integrity, cell cycle control, and cell apoptosis. Their roles in various processes of cancer progression, such as tumor invasion/metastasis, proliferation, and apoptosis/survival, as well as the roles in both cancer and cancer-associated cells, such as fibroblasts and endothelial cells, have been extensively explored (Chen et al. 2014; Kale et al. 2015; Mali et al. 2014; Matsuoka and Yashiro 2014; Morgan-Fisher et al. 2013; Rath and Olson 2012; Schofield and Bernard 2013). Although ROCK activation is generally considered to be oncogenic, some studies show that ROCK functions as a negative regulator in cancer progression. As a result, the precise role of ROCK in affecting different types of cancer process is context defined, specifically depending on cell type and the microenvironment surrounding a tumor.

### ROCK in Tumor Cell Invasion and Metastasis

The role of the Rho/ROCK pathway in tumor cell invasion and metastasis has been extensively studied since its role in promoting tumor cell dissemination in vivo was firstly reported (Itoh et al. 1999). Most studies favor a positive role of ROCK activation in enhancing tumor cell invasion and metastasis via direct effects on tumor cell motility and/or indirect effects on cancer-associated fibroblasts to increase extracellular matrix stiffness and facilitate cancer cell movement; inhibiting ROCK by chemical inhibitors leads to decreased tumor cell invasion and metastasis. Among the recent studies, Y27632 decreased breast cancer cell invasion/migration in vitro and metastasis in vivo in a mouse model of human breast cancer metastasis to human bone (Liu et al. 2009); fasudil or Y27632 decreased invasion and motility of CaOV3 and SKOV-3 ovarian cancer cell lines (Jeong et al. 2012; Ogata et al. 2009; Peng et al. 2012); fasudil suppressed the proliferation, migration, and invasion of A-549 lung cancer cells (Zhu et al. 2011); Y27632 decreased invasive potential of colon cancer SW620 cells (de Toledo et al. 2012); and Y27632 suppressed progression of hepatocellular carcinoma through both direct effects on the migration and proliferation of hepatocellular carcinoma and indirect effects on the pro-metastatic microenvironment by deactivating activated hepatic stellate cells in fatty liver (Mikuriya et al. 2015). The underlying mechanisms resulting from ROCK inhibition include reduction of stress fiber formation and peripheral focal adhesions (Liu et al. 2009), loss of membrane blebbing and re-established E-cadherin dependent adherent junctions (de Toledo et al. 2012), loss of intracellular cytoskeletal rearrangement (Ogata et al. 2009), inhibition of the epithelial to mesenchymal transition (Castro et al. 2013; de Toledo et al. 2012), and inhibition of proteolytic enzyme expression such as matrix metalloproteinase 9 and urokinase-type plasminogen activator (Jeong et al. 2012).

Contrasting to these studies demonstrating beneficial effects of ROCK inhibition, several others have shown the detrimental effects of ROCK inhibition. Y-27632 treatment activated dormant MCF-7 breast cancer cells through the disintegration of cell junctions coupled with the loss of E-cadherin and β-catenin from the cell membrane leading to increased migration and invasion in both two-dimensional and three-dimensional substrates (Yang and Kim 2014). Y27632 also increased the invasion of SW620 colon cancer cells in three-dimensional collagen matrix, but not in two-dimensional matrix (Vishnubhotla et al. 2012). In addition, treatment with Y-27632 in SW480 colon cancer cells also increased migration associated with dramatically altered focal adhesions (Adachi et al. 2011). Moreover, inhibition of ROCK2 through binding to coronin1A/B via its PH domain was required for neuregulin 1 stimulated scattering of MCF-7 cells (Rana and Worthylake 2012). Together, these studies reveal that the contribution of Rho/ROCK signaling to cancer cell migration varies depending on the cell line tested and on the surrounding microenvironment.

The contradicting effects of ROCK inhibition on tumor cell invasion and metastasis can be related to the great plasticity of cancer cells in their migratory mechanisms and to the activation of other signaling pathways involved in cell migration, for instance, Rac GTPase-mediated signaling (Fife et al. 2014; Kale et al. 2015; Li et al. 2015a; Matsuoka and Yashiro 2014; Prudnikova et al. 2015; Sadok and Marshall 2014; Yang and Kim 2014). Cancer cells are able to display different modes of motility which has been broadly classified as single-cell and collective-cell migration. Single-cell migration is further subdivided into elongated mesenchymal and rounded amoeboid types which have different requirements of molecular signaling. Cancer cells have also been shown to switch modes of migration after ROCK inhibition, for instance, from rounded amoeboid type to elongated mesenchymal type in Y27632 treated gastric cancer cells (Matsuoka et al. 2011). Drug combinations to simultaneously block several targets may produce greater anti-metastatic effects: combined inhibition of ROCK and Rac reduced mesenchymal motility of Y27632 treated gastric cancer cells (Matsuoka and Yashiro 2014; Matsuoka et al. 2011); combined inhibition of ROCK and myotonic dystrophy kinase-related Cdc42-binding kinases (MRCK) inhibited migration and invasion of lung, breast, melanoma, and pancreatic cancer cells (Kale et al. 2014, 2015). Finally, the recently developed new ROCK inhibitors with higher potency than Y27632 or fasudil may be more effective in blocking tumor cell invasion and metastasis (Sadok et al. 2015).

### ROCK in Tumor Cell Proliferation and Angiogenesis

Similar to its activity in tumor cell invasion and metastasis, a majority of studies support a positive role of ROCK in tumor growth through regulating cell proliferation and angiogenesis. Numerous reports indicate that ROCK inhibition decreases tumor cell proliferation and angiogenesis (Chen et al. 2014). Through its well-established role in promoting stress fiber formation, ROCK promotes fibronectin matrix assembly, cell adhesion, and colonization of metastatic kidney tumor cells (Knowles et al. 2015). Y-27632 treatment in melanoma significantly changed 94 gene transcripts, many of which are involved in tumor initiation and progression, indicating that ROCK signaling also contributes to the tumor transcriptome in addition to its well-established role in the regulation of F-actin dynamics (Spencer et al. 2011). In lung cancer, fasudil treatment inhibited the growth of 95D lung carcinoma cells (Yang et al. 2010); it also significantly attenuated angiogenesis as it inhibited lung carcinoma-conditioned endothelial cell proliferation and in vivo invasive ability by causing stress fiber fracture and breakage (Zhang et al. 2012). Moreover, ROCK inhibition may also decrease tumor cell proliferation by preventing the activation of oncogenes. ROCK phosphorylates the oncogene c-Myc at T58 and/or S62, ensuring higher stability and transcriptional activity of c-Myc in breast cancer (Liu et al. 2009) and in prostate cancer (Zhang et al. 2014); hence the inhibition of ROCK lowers c-Myc activity.

ROCK activity plays a critical role in cytokinesis in centrosomes, the microtubule organization centers governing chromosome segregation during cell division. Centrosome abnormality leads to genomic instability, representing a common feature of tumor cells. Extra centrosomes can result in aneuploidy and polyploidy, which are thought to be tumorigenic. At centrosomes, morgana/chp-1 directly binds ROCK2 and prevents ROCK2 activation by nucleophosmin; the down-regulation of morgana in mice or in patients with atypical chronic myeloid leukemia leads to increased ROCK2 kinase activity, which results in centrosome amplification and cytogenetic abnormalities (Di Savino et al. 2015; Ferretti et al. 2010). In breast cancer cells, BRCA2 directly binds to nucleophosmin and ROCK2 at centrosomes; the dysfunction of BRCA2, which accounts for the majority of heredity breast and ovarian cancer, causes aberrant centrosome amplification and a high frequency of multinucleated cells (Wang et al. 2011). While increased ROCK activity resulted in centrosome abnormality and genomic instability, ROCK inhibitors were also found to further increase chromosome instability and induce massive chromosome segregation errors and suppress T cell leukemia growth through inducing microtubule-dependent centrosome fragmentation (Oku et al. 2014).

Conversely, ROCK inhibition was also found to increase cell proliferation in other studies. In colon cancer (Nakashima et al. 2010) and pancreatic cancer cells (Nakashima et al. 2011), treatment with Y-27632 induced cell proliferation. These studies suggest that ROCK negatively regulates EGF-induced cell proliferation (Nakashima et al. 2010), while EGF first stimulates the activation of the EGF receptor and subsequently increases cancer cell proliferation, EGF concurrently induces the activation of ROCK, which then turns off the activated EGF receptor pathway via a negative feedback system. Thus, inhibiting ROCK with Y-27632 prevents the negative feedback, leading to increased EGF activity and cell proliferation (Nakashima et al. 2011). Furthermore, ROCK inhibition was reported to promote cell proliferation through the down-regulation of a tumor suppressor gene, phosphatase and tension homolog (PTEN), leading to the up-regulation of Akt phosphorylation which is essential for cell proliferation and survival (Fusella et al. 2014; Yang and Kim 2012). Taken together, ROCK has a general positive role in cancer cell proliferation in many cell types through promoting actomyosin cytoskeleton contractility and cell adhesion, cytokinesis, and activation of oncogenes; though there are some exceptions in specialized contexts through negative feedback on growth factor signaling (for instance, EGF) and promoting tumor suppressor gene activation (for instance, PTEN).

### ROCK in Tumor Cell Survival and Apoptosis

The investigations of the role of ROCK in tumor cell survival and apoptosis returned discordant marks. Many studies have shown that the inhibition of ROCK is beneficial through increasing apoptosis. For example, Y-27632 treatment of cultured melanoma cells decreased tumor cell invasion and altered cell survival; in addition, the treatment reduced melanoma tumor volume in tumor-bearing mice (Routhier et al. 2010). In bladder cancer, fasudil increased the apoptotic response (Abe et al. 2014). In leukemia, ROCK1 bound to Erk1/2, and inhibiting ROCK released Erk1/2, consequently increasing apoptosis (Li et al. 2013a). In acute myeloid leukemia (AML), ROCK1 depletion by genetic knockout or ROCK inhibition with H-1152, Y27632, or fasudil reduced the survival of malignant cells bearing oncogenic form of KIT, FLT3, and BCR-ABL through suppressing MLC phosphorylation (Mali et al. 2011). ROCK1 knockdown by siRNA or fasudil treatment also resulted in increased apoptosis and decreased viability of primary cells isolated from AML patients (Wermke et al. 2015).

Other investigations, in contrast, revealed that the inhibition of ROCK promotes tumor cell survival and chemoresistance, or reduces the apoptotic effects of anti-carcinogens. Y-27632 treatment in neuroblastoma increased cell survival and facilitated the development of chemoresistance to cisplatin due in part to altered expression of cisplatin resistance genes (Street et al. 2010). In leukemia, RhoA/ROCK1/PTEN activation was critical to apoptosis induced by R-(−)-gossypol acetic acid (AT-101), a natural cottonseed product that exhibits anti-cancer activity, but the treatment with Y27632, or down-regulation of ROCK1 with siRNA lowered the effectiveness of the small molecule inhibitor (Li et al. 2014b). Triptolide, an active component of a Chinese herbal remedy, induces apoptosis through caspase-3-mediated ROCK1 activation and MLC phosphorylation (Liu et al. 2013). Curcumin-induced apoptosis in ovarian cancer SKOV3 cells in part through activation of RhoA/ROCK pathway (Yin and Sun 2014). Taken together, these studies indicate that ROCK activation is anti-apoptotic in many cell types, especially in leukemia, by intervening oncogenic proliferation and survival signaling, whereas proapoptotic in other specific contexts, in particular in mediating apoptotic signaling triggered by some chemo-drugs or anti-carcinogens.

### ROCK in Cancer Stem Cells

Cancer stem cells, also named tumor-initiating cells, represent a small subpopulation of cancer cells and are recognized as the root cause behind cancer metastasis and recurrence. There has been a great deal of interest in expanding cancer stem cells in vitro for investigating their tumorigenicity. ROCK inhibition was initially observed to facilitate the in vitro growth of human embryonic stem cells by inhibiting dissociation-induced apoptosis, named anoikis, through the blockage of ROCK/MLC-regulated actomyosin contraction (Watanabe et al. 2007). Due to the prominent pro-survival effects of ROCK inhibition, the inclusion of ROCK inhibitors such as Y27632 has become part of standard stem cell culture protocols for embryonic, somatic, and cancer stem cells (Castro et al. 2013; Ohata et al. 2012; Tilson et al. 2015; Watanabe et al. 2007; Zhang et al. 2011). In addition to promoting survival, ROCK inhibitors also increase proliferation of cancer stem cells, for instance, mouse mammary epithelial tumor-initiating cells (Castro et al. 2013), and enhance stem-like phenotypes with increased expression of related stem cell markers (Ohata et al. 2012; Tilson et al. 2015). ROCK inhibition also cooperates with irradiated fibroblast feeder cells to induce conditional reprogramming of normal and tumor epithelial cells from various tissues into adult stem-like cells (Liu et al. 2012b; Palechor-Ceron et al. 2013; Suprynowicz et al. 2012), or into a progenitor cell-like phenotype (Saenz et al. 2014), capable of proliferating indefinitely in vitro. The mechanisms underlying the beneficial effects of ROCK inhibitors in stem cell culture in vitro are attributed to the blockade of actomyosin hypercontraction-mediated apoptosis (Castro et al. 2013; Ohata et al. 2012; Tilson et al. 2015; Watanabe et al. 2007; Zhang et al. 2011), the suppression of NOTCH signaling induced differentiation (Yugawa et al. 2013), and the limitation of epithelial to mesenchymal transition (Castro et al. 2013).

In contrast to the well-documented beneficial effects of ROCK inhibitors in promoting cancer stem cell expansion in culture, fewer studies have investigated the effects of ROCK inhibition on cancer stem cell tumorigenicity in vivo. Increasing ROCK-dependent contractility reduced adhesion of tumor-initiating cells from primary human glioblastoma to soft extracellular matrix leading to a rounded and immotile phenotype with reduced migration and tissue invasion (Wong et al. 2015). In contrast, RhoA/ROCK activation in stromal cells surrounding cancer stem cells increased stiffness of extracellular matrix leading to increased stem cell adhesion to extracellular matrix and consequently increased spreading, migration, and proliferation (Choi et al. 2015; Wong et al. 2015). Similar to the activation of dormant MCF-7 breast cancer cells by ROCK inhibition as mentioned above (Yang and Kim 2014), ROCK inhibition in cancer stem cells may alter the activation of other signaling pathways involved in regulation of cytoskeleton, for instance, Rac and Cdc42 GTPase-mediated signaling activation, therefore increasing cytoskeletal plasticity and cell adhesion to extracellular matrix, and integrin-mediated signaling. These ROCK inhibition-related potential pro-survival and pro-extracellular matrix adhesive effects on cancer stem cells and on dormant cancer cells represent a risk for cancer cell dissemination and metastasis, and therefore need to be considered to avoid the potential undesirable effects of ROCK inhibition therapy.

### Overlapping and Differential Roles of ROCK Isoforms in Tumorigenesis

Current research on ROCK function and its clinical applications as a potential therapeutic target are mainly dependent on the use of small molecule inhibitors; however, these inhibitors modulate the activity of both ROCK proteins. One possible explanation for the apparently inconsistent roles of ROCK in various cancer processes is that the two ROCK isoforms have both overlapping and unique functions, which can even oppose one another in specialized contexts. We have recently shown the distinct roles of the ROCK isoforms in mouse embryonic fibroblasts in regulating the actin cytoskeleton under stress conditions; ROCK1 is involved in MLC phosphorylation, actomyosin contraction, and disruption of central stress fibers, while ROCK2 stabilizes the actin cytoskeleton through cofilin phosphorylation (Shi et al. 2013a, b; Surma et al. 2014; Wei et al. 2015). Other recent studies have also revealed functional differences between ROCK1 and ROCK2 in regulating the actin cytoskeleton and other cellular functions in non-tumor cells (Chun et al. 2012; Herskowitz et al. 2013; Lock et al. 2012; Newell-Litwa et al. 2015).

In tumor cells, ROCK1 and ROCK2 have recently been reported to have functional differences in regulating adhesion, migration, proliferation, and gene expression, but the underlying mechanisms are not fully understood (Inaba et al. 2010; Mertsch and Thanos 2014; Montalvo et al. 2013; Rochelle et al. 2013; Vega et al. 2011; Vigil et al. 2012; Wang et al. 2014). In non-small cell lung carcinoma, suppression of ROCK1 or ROCK2 expression alone was sufficient to impair anchorage-independent growth (Vigil et al. 2012). A decreased cell migration rate was observed with either ROCK1 or ROCK2 knockdown in mouse pancreatic endothelial cells and angiosarcoma cells, only ROCK2 knockdown showed reduced phosphorylation of MLC phosphatase and cofilin (Montalvo et al. 2013). In retinoblastoma cells, inhibiting ROCK1 with siRNA increased adhesion and decreased invasive capacity similarly to ROCK inhibition by Y27632, but no effect was observed after inhibiting ROCK2 by siRNA, possibly due to a lower expression of ROCK2 than ROCK1 in these cells (Wang et al. 2014). In glioblastoma cells, knocking down ROCK1 by shRNA impaired cell migration and reduced cell proliferation similarly to ROCK inhibition by Y27632, while in contrast, ROCK2 knockdown increased cell migration and proliferation (Mertsch and Thanos 2014). In these cells, ROCK1 knockdown also reduced ROCK2 expression, and not conversely; therefore, ROCK1 knockdown may result in a greater reduction of total ROCK activity than ROCK2 knockdown (Mertsch and Thanos 2014). Together, these studies suggest that inhibition of one ROCK isoform in tumor cells may inhibit tumorigenicity similarly to ROCK pan-inhibition, but with less induction of other signaling pathways involved in cytoskeleton regulation, therefore reducing the potential undesirable effects of ROCK inhibition therapy as reviewed above. The functional differences for ROCK1 and ROCK2 in tumor and non-tumor cells could be explained by their variations in expression levels, subcellular locations, and interaction partners in individual cell types (Amano et al. 2010a; Morgan-Fisher et al. 2013; Schofield and Bernard 2013; Shi and Wei 2007). More investigations on ROCK isoform function in cancer are required in order to elucidate the underlying mechanisms of their functional differences and determine the predominant functional isoform in the relevant tumor types.

## Promising Potential of ROCK Inhibition in Cancer Therapy

The most commonly used chemical inhibitors of ROCK are fasudil (also named HA-1077) (Asano et al. 1987) and Y27632 (Uehata et al. 1997). Fasudil is the only ROCK inhibitor used in humans for systemic applications, and was approved in Japan in 1995 for the prevention and treatment of cerebral vasospasm after surgery in subarachnoid hemorrhagic patients (Shibuya et al. 1992). Hydroxyfasudil is the main metabolite of fasudil after oral administration, and H-1152P is another analog of fasudil, both of them are more potent than fasudil. Because these inhibitors target the ATP-dependent kinase domain of ROCK1 and ROCK2, they are non-isoform specific and also inhibit other serine/threonine kinases such as PKA and PKC at higher concentrations (Bain et al. 2007; Davies et al. 2000). These inhibitors have shown beneficial effects in a variety of animal disease models including cardiovascular, metabolic, neurodegenerative, and inflammatory diseases, along with various types of cancer (Fukumoto and Shimokawa 2013; Huang et al. 2013; Knipe et al. 2015; Morgan-Fisher et al. 2013; Sawada and Liao 2014; Shi and Wei 2013; Watzlawick et al. 2014). Due to the overall promising results of ROCK inhibition, considerable interest and efforts have been devoted to the development of more potent and selective ROCK inhibitors (Feng and LoGrasso 2014; Guan et al. 2013) including non-isoform selective (Tables 3, 4) and isoform selective inhibitors (Table 5). Among these novel ROCK inhibitors, ripasudil (also named K-115), a close analog of fasudil, has recently reached the stage of clinical application: it was approved in Japan in 2014 for the treatment of glaucoma (Garnock-Jones 2014).

**Table 3:** Novel ROCK inhibitors and potential therapeutic implications

Compound	Isoform selectivity	Therapeutic Implications	Species	References^a^
SAR-407899	IC_50_ 276 nM for ROCK1, IC_50_ 102 nM for ROCK2	Hypertension	Rats, human tissue	Grisk et al. (2012), Lohn et al. (2009)
		Erectile dysfunction	Rats, rabbits	Guagnini et al. (2012)
			Phase 2 trial	NCT00914277
		Chronic kidney disease	Mice	Babelova et al. (2013)
			Phase 1 trial	NCT01485900
Azaindole-1	*K*_i_ 3.7 nM for ROCK1, *K*_i_ 4.8 nM for ROCK2	Pulmonary hypertension	Rats	Pankey et al. (2012)
		Erectile dysfunction	Rats	Lasker et al. (2013)
FSD-C10		Encephalomyelitis	Mice	Li et al. (2014c)
DW1865	IC_50_ 100 nM for ROCK1, IC_50_ 20 nM for ROCK2	Hypertension	Rats	Oh et al. (2013b)
DL0805	IC_50_ 6.67 µM for ROCK1	Hypertension	Rats	Gong et al. (2012)
AMA 0076	IC_50_ 3.7 nM for ROCK1, IC_50_ 2.3 nM for ROCK2	Glaucoma	Rabbits	Van de Velde et al. (2014)
			Phase 1 trial	NCT02003547
			Phase 2 trial	NCT02136940 NCT01693315
K-115 (Ripasudil)	IC_50_ 51 nM for ROCK1, IC_50_ 19 nM for ROCK2	Glaucoma	Mice, rabbits, monkeys	Isobe et al. (2014), Yamamoto et al. (2014)
			Phase 1 trial	Tanihara et al. (2013a)
			Phase 2 trial	Tanihara et al. (2013b)
			Approved (Japan)	Garnock-Jones (2014)
AR-12286		Glaucoma	Phase 1 trial	Kopczynski et al. (2013) NCT01250197
			Phase 2 trial	Williams et al. (2011) NCT01330979, NCT01060579, NCT01302249, NCT01474135, NCT01789736, NCT00902200, NCT01699464, NCT02152774, NCT02173223
AR-13324		Glaucoma	Rabbits, monkeys	Kiel and Kopczynski (2015), Wang et al. (2015)
			Phase 1 trial	Levy et al. (2015) NCT01997879
			Phase 2 trial	Bacharach et al. (2015) NCT02057575, NCT01528787, NCT01731002
			Phase 3 trial	NCT02207621, NCT02246764

Only some most recent studies are included due to space limitation

^a^Clinical trial identifier numbers can be found in https://clinicaltrials.gov

**Table 4:** ROCK inhibitors and potential therapeutic implications in cancers

Compound	Inhibitory activity	Therapeutic implications	Species	References
Fasudil	*K*_i_ 0.4 µM for ROCK	Leukemia	Human cells, mice	Mali et al. (2011), Wermke et al. (2015)
		Brain cancer	Human cells, mice	Nakabayashi and Shimizu (2011)
		Lung cancer	Human cells	Zhu et al. (2011)
		Ovarian cancer	Human cells, mice	Ogata et al. (2009)
		Liver cancer	Human cells	Takeba et al. (2012)
		Bladder cancer	Human cells	Abe et al. (2014)
Y-27632	*K*_i_ 0.22 µM for ROCK1, *K*_i_ 0.30 µM for ROCK2	Leukemia	Human cells, mice	Burthem et al. (2007), Mali et al. (2011)
		Breast cancer	Human cells, mice	Liu et al. (2009), Yang and Kim (2014)
		Melanoma	Human cells, mice	Routhier et al. (2010)
		Prostate cancer	Human cells, mice	Zhang et al. (2014)
		Ovarian cancer	Human cells	Jeong et al. (2012)
H-1152	*K*_i_ 1.6 nM for ROCK	Breast cancer	Human cells, mice	Castro et al. (2013)
PT262	IC_50_ 5 µM for ROCK	Lung cancer	Human cells	Tsai et al. (2011)
RKI-1447	IC_50_ 14.5 nM for ROCK1, IC_50_ 6.2 nM for ROCK2	Breast cancer	Human cells	Patel et al. (2012)
RKI-18	IC_50_ 397 nM for ROCK1, IC_50_ 349 nM for ROCK2	Breast cancer	Human cells	Patel et al. (2014)
OXA-06	IC_50_ 5 nM for ROCK	Lung cancer	Human cells	Vigil et al. (2012)
DJ4	IC_50_ 5 nM for ROCK1, IC_50_ 50 nM for ROCK2	Lung cancer, melanoma, pancreatic cancer, breast cancer	Human cells	Kale et al. (2014)
AT13148	IC_50_ 6 nM for ROCK1, IC_50_ 4 nM for ROCK2	Melanoma	Human cells, mice	Sadok et al. (2015)
		Phase 1 trial	Breast, prostate, ovarian cancer	NCT01585701
CCT129254	IC_50_ 214 nM for ROCK1, IC_50_ 141 nM for ROCK2	Melanoma	Human Cells, mice	Sadok et al. (2015)

Only some most recent studies are included due to space limitation

**Table 5:** ROCK isoform selective inhibitors and potential therapeutic implications

Compound	Isoform specificity	Therapeutic implications	Species	References^a^
MBPTA (ROCK1)	IC_50_ 8.68 µM for ROCK1, IC_50_ 203.2 µM for ROCK2	Parkinson’s disease	Human cells	Chong et al. (2014)
KD-025/SLx-2119 (ROCK2)	IC_50_ 24 µM for ROCK1, IC_50_ 0.105 µM for ROCK2	Cerebral ischemia	Mice	Lee et al. (2014a)
		Rheumatoid arthritis	Human cells	Zanin-Zhorov et al. (2014)
		Psoriasis vulgaris	Phase 1 trial	NCT02106195
			Phase 2 trial	NCT02317627
Compound 24 (ROCK2)	IC_50_ 1.69 µM for ROCK1, IC_50_ 0.1 µM for ROCK2		Human cells	Li et al. (2012)
SR3677 (ROCK2)	IC_50_ 56 nM for ROCK1, IC_50_ 3 nM for ROCK2		Porcine tissue	Feng et al. (2008)
CID5056270 (ROCK2)	IC_50_ 13 nM for ROCK1, IC_50_ 0.56 nM for ROCK2		Human cells	Pireddu et al. (2012)

^a^Clinical trial identifier numbers can be found on https://clinicaltrials.gov

### Development of New ROCK Pan-Inhibitors and Isoform Selective Inhibitors

The resolution of the crystal structures of ROCK1 complexes with four different ATP-competitive inhibitors (Y-27632, fasudil, hydroxyfasudil, and H-1152P) is constructive for developing highly selective and more potent inhibitors (Jacobs et al. 2006). Assisted by structure-guided design, various screening methods have been employed toward the identification of novel inhibitors, including high-throughput library screening (Oh et al. 2013a), structure-guided and fragment-based screening, which uses small molecules to represent fragments rather than entire molecules to find possible molecular interactions (Li et al. 2012), and virtual screening using a computer-aided drug design strategy (Gong et al. 2010; Mishra et al. 2014; Shen et al. 2013). Because most of the recently developed novel inhibitors are still targeting the ROCK ATP pocket, they are generally not isoform selective (Tables 3, 4).

Efforts have recently been devoted to the development of ROCK isoform selective inhibitors. Several compounds with significant selectivity for ROCK2 over ROCK1 have been reported (Boerma et al. 2008; Feng et al. 2008; Li et al. 2012) (Table 5). SLx-2119, (also named KD-025), exhibits IC_50_ values of 0.105 µM for ROCK2 and 24 µM for ROCK1, though it is also an ATP-competitive inhibitor (Boerma et al. 2008). A combined approach using high concentration biochemical assays and fragment-based screening assisted by structure-guided design has discovered a novel series of ROCK inhibitors, in which compound 24 possessed more specificity against ROCK2 (IC_50_ values of 0.1 µM) over ROCK1 (IC_50_ values of 1.69 µM) (Li et al. 2012). SR3677 was also more selective for ROCK2 (IC_50_ values of ~3 nM) over ROCK1 (IC_50_ values of 56 nM) (Feng et al. 2008).

Novel ROCK inhibitors have been used to study non-cancer diseases (Tables 3, 5). A benzofuran derivative MBPTA has shown neuroprotective effects in SH-SY5Y neuroblastoma cells (Chong et al. 2014). SAR-407899 improved erectile dysfunction in rats and rabbits (Guagnini et al. 2012) and inhibited renal failure progression in mice with kidney disease (Babelova et al. 2013). Azaindole-1 improved erectile dysfunction (Lasker et al. 2013) and promoted vasodilation in pulmonary hypertensive rats (Pankey et al. 2012). SLx-2119 (KD-025), a highly ROCK2 specific inhibitor, was tested in mice as a treatment for cerebral ischemia (Lee et al. 2014a). FSD-C10 is a fasudil derivative and intranasal deliverable, and it exhibited effects on suppressing experimental autoimmune encephalomyelitis and promoting neuroprotection in mice (Li et al. 2014c). Other studies have reported blood pressure lowering effects of DW1865 in hypertensive rats (Oh et al. 2013b), vasorelaxant effects of DL0805 in isolated rat thoracic aorta (Gong et al. 2012), and intraocular pressure lowering effects of AMA 0076 (Van de Velde et al. 2014) and K115 (Isobe et al. 2014) in rabbits and monkeys as a treatment for glaucoma.

### ROCK Inhibitors Broadly Used in Experimental Cancer Studies

ROCK inhibitors fasudil and Y27632 have been extensively used in studies using cancer cell lines and rodent cancer models, and significant beneficial effects have been shown in many types of cancers (Chen et al. 2014; Kale et al. 2015; Mali et al. 2014; Matsuoka and Yashiro 2014; Morgan-Fisher et al. 2013; Rath and Olson 2012; Schofield and Bernard 2013) (Table 4). Recent experimental studies have further supported fasudil as a drug candidate for hematological malignancies (Mali et al. 2011; Oku et al. 2014; Wermke et al. 2015), lung cancers (Yang et al. 2010, 2012; Zhang et al. 2012; Zhu et al. 2011), bladder cancer (Abe et al. 2014), glioma (Nakabayashi and Shimizu 2011), hepatocellular carcinoma (Takeba et al. 2012), and ovarian cancer (Ogata et al. 2009). Several novel ROCK inhibitors were tested as an anti-cancer therapy (Table 4). PT262 caused cytoskeleton remodeling and was more effective than Y27632 or H-1152 in inhibiting migration of A549 lung carcinoma cells (Tsai et al. 2011). RKI-1447 and RKI-18 prevented breast cancer cell migration and invasion, and anchorage-independent colony formation (Patel et al. 2012, 2014). OXA-06 was used to show that anchorage-independent growth and matrigel invasion of non-small cell lung carcinoma cells were ROCK dependent (Vigil et al. 2012). DJ4, a multi-kinase inhibitor of both ROCK and MRCK, inhibited migration and invasion of lung, breast, melanoma, and pancreatic cancer cells (Kale et al. 2014). CCT129254 and AT13148, discovered as ATP-competitive AKT kinase inhibitors, also potently inhibited both ROCK1 and ROCK2 activity leading to a collapsed cytoskeletal phenotype, which was not observed in cells treated with less potent inhibitors Y27632 or H1152 (Sadok et al. 2015). The potent inhibition of actomyosin contraction by CCT129254 and AT13148 dramatically impaired melanoma cell invasion in culture, and reduced metastasis of melanoma cells in vivo (Sadok et al. 2015). As a result of more potent and selective ROCK inhibitors becoming available, more experimental studies and new screening strategy are underway to evaluate their potential use in cancer therapy.

### Combinations of ROCK Inhibition with Other Anti-Cancer Therapies

ROCK inhibition has also been examined as a possible augmentation to current chemotherapy treatments. In chronic myeloid leukemia, Y27632 and fasudil added to the anti-proliferative and pro-apoptotic effects of imatinib, a tyrosine kinase inhibitor (Burthem et al. 2007; Di Savino et al. 2015). In ovarian cancer, fasudil and Y27632 enhanced the anti-cancer drug cisplatin efficacy in inhibiting growth and inducing apoptosis of human cancer cell lines (Ohta et al. 2012). In malignant glioma, ROCK1 knockdown by shRNA increased the efficacy of nimustine hydrochloride, an alkylating drug used for glioma patients in Japan (Inaba et al. 2010). Also in glioma, ROCK2 siRNA worked synergistically with the anti-cancer drug temozolomide, increasing the induction of apoptosis and inhibiting the migration of U251 cells (Wen et al. 2014). In NRAS mutant melanoma, simultaneous inhibition of MEK and ROCK by anti-cancer drug trametinib and fasudil-induced apoptosis and suppressed growth of established tumors, at concentrations where the single drugs had little effect (Vogel et al. 2015). These studies reveal that a combined therapeutic stratagem of ROCK inhibitors with classic or new anti-cancer drugs might provide greater anti-cancer effects while reducing chemoresistance and side effect.

However, as mentioned above, combining ROCK inhibition with chemotherapeutic agents may lead to enhanced tumor chemoresistance in some circumstances. For example, ROCK inhibition by Y27632 in neuroblastoma cells resulted in enhanced tumor survival following cisplatin cytotoxicity (Street et al. 2010). In addition, ROCK inhibition reduced the favorable effects of several natural anti-carcinogens: indole-3-carbinol in inhibiting motility in breast cancer cells (Brew et al. 2009); curcumin in inducing apoptosis in ovarian cancer cells (Yin and Sun 2014); R-(−)-gossypol acetic acid (Li et al. 2014b); and triptolide (Liu et al. 2013) in inducing apoptosis in leukemia cells. Hence these detrimental effects of ROCK inhibitors need to be considered and should be ruled out while developing novel anti-cancer therapies incorporating ROCK inhibitors.

### Clinical Implications of ROCK Inhibitors for Cancers and Non-Cancer Diseases

Despite the significant promise achieved from experimental studies of merging ROCK inhibition into therapeutic strategies, there is only one reported clinical trial using ROCK inhibitors in cancer treatment: AT13148 in phase 1 clinical trial initiated in 2012 for the treatment of advanced solid tumors (ClinicalTrials.gov identifier NCT01585701). AT13148 also inhibits several members of AGC kinase family including AKT and PKA (Sadok et al. 2015). Of the four most used ROCK inhibitors (Y-27632, fasudil, hydroxyfasudil, and H-1152P), fasudil is the only one approved for the treatment of vasospasm after subarachnoid hemorrhage (Shibuya et al. 1992), and has been used in clinical studies primarily for the treatment of cardiovascular diseases (Shi and Wei 2013). Current clinical trials for fasudil found on ClinicalTrials.gov include atherosclerosis (NCT00120718, NCT00670202), Reynaud’s phenomenon (NCT00498615), diabetes complications (NCT01823081), and neuronal disease (NCT01935518). Clinical trials using novel ROCK inhibitors have mainly been conducted for cardiovascular disease and eye disease (Tables 3, 5). SAR-407899 has been used to treat kidney disease (NCT01485900) and erectile dysfunction (NCT00914277). SLx-2119 (KD-025) has been used to treat psoriasis vulgaris (NCT02106195) and examines the response in autoimmune disease (Zanin-Zhorov et al. 2014). Several clinical studies have been performed to treat glaucoma, ocular hypertension, and eye disease including AMA0076 in phase 1 (NCT02003547) and phase 2 trials (NCT01693315, NCT02136940), AR-12286 in phase 1 (Kopczynski et al. 2013) (NCT01250197) and phase 2 trials (Williams et al. 2011) (NCT01330979, NCT01060579, NCT01302249, NCT01474135, NCT01789736, NCT00902200, NCT01699464, NCT02152774, NCT02173223), AR-13324 in phase I (Levy et al. 2015) (NCT01997879) and phase 2 trials (Bacharach et al. 2015) (NCT02057575, NCT01528787, NCT01731002). After phase 3 clinical trials, Ripasudil (K-115) was approved for glaucoma in Japan in 2014 (Garnock-Jones 2014). Given the accumulated indexes of safety and efficiency from fasudil and some novel ROCK isoform-specific inhibitors in clinic trials, the application prospect of anti-cancer drugs targeting ROCK is exhilarating; we expect ROCK inhibitors will become valuable anti-cancer members in the near future and will make contributions to reducing tumor growth, decreasing metastasis, and improving disease outcome.

## Conclusions and Future Direction

The ROCK proteins contribute to a broad range of cellular functions with their main impact on the regulation of many cytoskeletal-associated proteins. Accumulating evidences have been showing that ROCK signaling is critically responsible, at least in part, for many important cancer-associated phenotypes. There is an increasing interest in targeting ROCK signaling in cancer therapeutics, especially in obstructing tumor cell invasion, metastasis, tumor growth, angiogenesis, cancer-associated alterations of extracellular matrix, and hematological malignancies. Although the ROCK inhibitor fasudil has been available in clinical application for 20 years, it has not yet been used in cancer treatment, and the number of clinical trials for human cancer is still limited.

There are a number of challenges in translating updating knowledge of ROCK signaling into anti-cancer therapy; the current information on the role of ROCK signaling in tumorigenicity is still incomplete, in particular, the mechanisms underlying both the positive and negative roles of ROCK in regulating migration, proliferation, apoptosis/survival, and chemoresistance of tumor cells, including primary tumor cells, tumor stem cells, and dormant tumor cells, remain unclarified. Nevertheless, it has become progressively clear that the consequences of ROCK inhibition and the induction of compensatory signaling pathways, especially those involving in cytoskeleton regulation, largely depend on the tumor cell type, cell context, and the microenvironment; therefore, the complexity in evaluating the application of ROCK inhibitors is augmented. Combined inhibition of ROCK and compensatory signaling could be useful to avoid potential undesirable effects of ROCK inhibition. Furthermore, the first generation of ROCK inhibitors including fasudil shows non-specific inhibitory effects on other kinases, so it may cause off-target effects. Many more potent and selective ROCK inhibitors have recently been developed, some of them may show promising potential in cancer therapy in the near future. It is worth noting that the combination therapy of ROCK inhibitors and other anti-cancer drugs produces greater anti-cancer effects while resistance to single agent is reduced.

There is increased agreement that ROCK1 and ROCK2 have both redundant and non-redundant functions, and isoform-specific inhibition may therefore elicit different biological effects. Although the majority of currently available ROCK inhibitors are suitable initial tools for investigating the role of ROCK in cancer, their limitation in mechanistic studies is unavoidable due to their non-isoform selective nature. Additionally, ROCK1 and ROCK2 expression and/or activity can be separately regulated by numerous factors, which either positively or negatively modify their catalytic activity and/or subcellular localization. Moreover, there are an increased number of somatic mutations and miRNAs being discovered and evaluated for their differing impacts on ROCK isoform expression and activity. Hence, in order to develop isoform-specific targeting strategy for cancer therapy, it is necessary to understand the different functions of each ROCK isoform in cancer pathophysiology and ascertain the major functional isoform in specific tumor types; consequently, it will reduce toxic and undesirable effects than pan-inhibition in clinic application. Recently developed ROCK1 and ROCK2 isoform-specific genetically modified mouse models have offered a unique opportunity to analyze in vivo physiological and pathological functions of ROCK1 and ROCK2 (Lee et al. 2014b; Rikitake et al. 2005; Shi et al. 2011; Shimizu et al. 2005, 2013; Soliman et al. 2015; Thumkeo et al. 2003; Zhang et al. 2006), including cancer development and progression (Mali et al. 2011; Samuel et al. 2011). Some novel isoform selective inhibitors are becoming commercially available and will serve as valuable tools for further dissecting the roles of ROCK1 and ROCK2 in cancer and other diseases. Importantly, the continuation of significant progress in basic and preclinical researches will undoubtedly move ROCK isoform inhibitors to future clinical practice.

## Abbreviations

ROCK: Rho-associated coiled-coil kinase
PKA: Protein kinase A
PKG: Protein kinase G
PKC: Protein kinase C
RBD: Rho-binding domain
PH: Pleckstrin homology
MLC: Myosin light chain
siRNA: Short interfering RNA
shRNA: Short hairpin RNA
miRNA: MicroRNA
EGF: Epidermal growth factor
